# Role of mTOR, Bad, and Survivin in RasGAP Fragment N-Mediated Cell Protection

**DOI:** 10.1371/journal.pone.0068123

**Published:** 2013-06-27

**Authors:** Nieves Peltzer, Güliz Vanli, Jiang-Yan Yang, Christian Widmann

**Affiliations:** Department of Physiology, University of Lausanne, Lausanne, Switzerland; H. Lee Moffitt Cancer Center & Research Institute, United States of America

## Abstract

Partial cleavage of p120 RasGAP by caspase-3 in stressed cells generates an N-terminal fragment, called fragment N, which activates an anti-apoptotic Akt-dependent survival response. Akt regulates several effectors but which of these mediate fragment N-dependent cell protection has not been defined yet. Here we have investigated the role of mTORC1, Bad, and survivin in the capacity of fragment N to protect cells from apoptosis. Neither rapamycin, an inhibitor of mTORC1, nor silencing of raptor, a subunit of the mTORC1 complex, altered the ability of fragment N from inhibiting cisplatin- and Fas ligand-induced death. Cells lacking Bad, despite displaying a stronger resistance to apoptosis, were still protected by fragment N against cisplatin-induced death. Fragment N was also able to protect cells from Fas ligand-induced death in conditions where Bad plays no role in apoptosis regulation. Fragment N expression in cells did neither modulate survivin mRNA nor its protein expression. Moreover, the expression of cytoplasmic survivin, known to exert anti-apoptotic actions in cells, still occurred in UV-B-irradiated epidermis of mouse expressing a caspase-3-resistant RasGAP mutant that cannot produce fragment N. Additionally, survivin function in cell cycle progression was not affected by fragment N. These results indicate that, taken individually, mTOR, Bad, or Survivin are not required for fragment N to protect cells from cell death. We conclude that downstream targets of Akt other than mTORC1, Bad, or survivin mediate fragment N-induced protection or that several Akt effectors can compensate for each other to induce the pro-survival fragment N-dependent response.

## Introduction

Activation of executioner caspases was once believed to represent a point of no return in the path to death. However it is now well established that while executioner caspases are indispensable for apoptosis, there are situations when their activation does not lead to death. For example, healthy dividing cells can weakly activate caspase-3 in response to mild stresses [1]. Caspase-3 also participates, in an apoptosis-independent manner, in T and B cell homeostasis [2], [3], in microglia activation [4], and in muscle [5], monocyte [6], bone marrow stromal stem cell [7], and erythroid cell differentiation [8].

Low caspase-3 activation in stressed cells induces the partial cleavage of RasGAP into an amino-terminal fragment, called fragment N, that prevents amplification of caspase-3 activation and death in an Akt-dependent manner [9]. Knock-in mice that only express a caspase-3-resistant RasGAP mutant, and hence that cannot generate fragment N in response to stress, are unable to stimulate Akt efficiently and are more sensitive to damage induced by various pathophysiological insults [10]. Fragment N generation can therefore explain why cells having mildly activated caspase-3 do not necessarily die. On the other hand, when caspase-3 activity is strongly stimulated in cells, fragment N is further processed into smaller fragments, called N1 and N2, that no longer have the capacity to activate Akt [11]. The extent of caspase-3 activity in a cell can therefore be sensed by RasGAP to either mount an efficient Akt-dependent protection when the stress is not too strong [1], [12] or to abrogate this protective signal in cells faced with strong insults or apoptotic stimuli [11].

Phosphorylation of downstream effectors by Akt leads to diverse cellular responses affecting metabolism, protein synthesis, proliferation, angiogenesis and inhibition of apoptosis [13]. A series of Akt effectors have been shown to favor survival once phosphorylated by Akt [13], [14]. These can be either pro-apoptotic proteins that become inactivated once phosphorylated by Akt or anti-apoptotic proteins the expression of which is induced by Akt. In the first group lie Bad, Bax, Ask1, and pro-apoptotic transcription factors such as YAP and some Forkhead family members. Mdm2, a p53 inhibitor, members of the inhibitor of apoptosis family (c-IAP1/2, survivin), members of the anti-apoptotic Bcl-2 subfamily (A1, Bcl-X_L_) and the NF-kB transcription factor, which is generally inducing survival responses, are found in the second group [15]–[18].

Although Akt can lead to activation of the NF-κB transcription factor, fragment N-mediated Akt activation does not result in NF-κB stimulation [19]. In fact, fragment N is able to block NF-κB activation in response to various stimuli including exposure to inflammatory cytokines [20], [21]. Hence fragment N does not rely on NF-κB activation to protect cells. Actually, a sustained NF-κB activation could be detrimental at least in certain cell types, such as pancreatic beta cells [22]. In this context, the capacity of fragment N to block NF-κB activation would be beneficial and it has indeed been shown that NF-κB inhibition by fragment N contributes to its anti-apoptotic activity in beta cells [21], [23]. These observations rule out activation of NF-κB as an Akt-dependent mechanism used by fragment N to protect cells. Which of the other Akt effectors are required for fragment N to protect cells is not known.

In the present work we investigated whether mTORC1, Bad and survivin play a role in fragment N-mediated apoptosis inhibition. Mammalian TOR (mTOR) is a protein kinase that exists in two different complexes. The mTORC1 complex contains mTOR and Raptor and is inhibited by rapamycin. Akt indirectly activates mTORC1 by preventing the TSC1/TSC2 GTPase-activating proteins from inhibiting Rheb, the small GTP-binding protein that stimulates mTORC1. The mTORC2 complex, which contains mTOR and Rictor, is not sensitive to rapamycin, at least in short-term experiments [24], and positively regulates the activation of Akt by phosphorylating it rather than being an Akt effector itself [25], [26]. The involvement of mTORC1 in the control of cell survival responses [27], [28] makes it a possible fragment N effector candidate.

Another putative player in fragment N-induced protective pathway is Bad, a pro-apoptotic member of the Bcl-2 family [29], [30]. When phosphorylated by Akt, Bad binds to 14-3-3 proteins and this prevents its capacity to interact with anti-apoptotic Bcl-2 family members. Consequently, phosphorylated Bad loses its pro-apoptotic activity [31].

Survivin (also called BIRC5), an atypical member of the inhibitor of apoptosis (IAP) family of proteins, is also a component of the chromosomal passenger protein complex that ensures proper segregation of sister chromatids and cytokinesis [32], [33], and that mediates many other functions during mitosis [34]. The absence of survivin profoundly alters mitosis and cytokinesis and this eventually leads to cell death [32], [35]. The exact contribution of survivin in anti-apoptotic responses is controversial and still not well defined. Survivin may indirectly affects caspases either by binding and stabilizing XIAP [36], blocking Smac-dependent XIAP inhibition [19], [24], [30], [33] or inhibiting caspase-9 by cooperating with HBXIP [23]. Depending on the cell types and the experimental conditions, activation of Akt can lead to increased survivin expression [37], [38].

## Materials and Methods

### Plasmids

**pEGFP-C1** (#6) encodes the green fluorescent protein and is from Clontech. The.dn3,.lti,.gl3, and.ebg extensions indicate that the backbone plasmid is pcDNA3 (Invitrogen), a lentiviral vector, pGL3 basic (Promega) and pEBG (Addgene), respectively. **HA-hRasGAP[1-455](D157A).dn3** (#352) encodes a tagged and caspase-3-resistant version of the 1–455 amino acid fragment of human RasGAP (fragment N); previously called N-D157A.dn3 in [12]. **HA-hRasGAP[1–455](D157A).lti** (#353) is the plasmid used for the production of the lentivirus encoding the caspase-resistant form of fragment N; previously called N-D157A.lti in [1]. **GFP-HA-hRasGAP[1–157]** (#231) encodes a fusion protein between GFP and fragment N1 (amino acid 1–157 of human RasGAP). It was generated by subcloning the ApaI/HindIII fragment of plasmid HA-hRasGAP[1–158].dn3 (#147; previously called HA-N1.dn3 in [12]) into pEGFP-C3 (#98; Clontech) opened with the same enzymes. **GFP-HA-hRasGAP[157–455]** (#213), previously described as GFP-HA-N2 in [39], encodes a fusion protein between GFP and fragment N2 (amino acid 158–455 of human RasGAP). **GST-mBad.ebg** (#271) is a mammalian expression vector encoding a fusion protein between glutathione S-transferase (GST) and full-length murine Bad. It was purchased from Cell Signaling under the name pEBG-MBad. **SIN-PGK-hBcl2-WHV** (#359) was used for the production of Bcl2-encoding lentiviruses [23]. **mE2F1.sport6** (#675) encodes the mouse E2F1 transcription factor. It was purchased from Imagenes. Mouse survivin promoter plasmids were obtained by cloning the sequences corresponding to the entire survivin promoter (−1297 to +13) or a shorter sequence containing the minimal sequences required for its activation (−209 to +13) [40], [41] into the pGL3 basic promoterless luciferase gene-bearing plasmid (#95). The resulting plasmids were called **mSurvivin promoter [**−**1297 to +13].gl3** (#634) and **mSurvivin promoter [**−**209 to +13].gl3** (#633), respectively. mSurvivin promoter [−1297 to +13].gl3 was generated as follows. The genomic DNA of the FVB/N-tg(RIP::N)1Wid mouse [20] (European Mouse Mutant Archive accession n°EM:05139) was PCR-amplified using the sense primer #655: AAAAAA (feeder) CTCGAG (XhoI) CACCTCTTACTCCACACC TG (nucleotides 49001–49020 of the mouse chromosome 11 genomic contig NT_165773) and the anti-sense primer #656: AAAAAA (feeder) AAGCTT (HindIII) CCG GAG CTC CCA TGA TGG CG (nucleotides 50310-50291 of the mouse chromosome 11 genomic contig NT_165773). The resulting 1134 base pair fragment was cut with XhoI and HindIII and sub-cloned in pGL3-basic (#95) opened with the same enzymes. mSurvivin promoter [−209 to +13].gl3 was generated as follows. Plasmid mSurvivin promoter [−1297 to +13].gl3 (#634) was PCR-amplified using the sense primer #657: AAAAAA (feeder) CTCGAG (XhoI) ATGCCCTGCGCCCGCCACGC (nucleotides 50089–50108 of the mouse chromosome 11 genomic contig NT_165773) and the anti-sense primer #656 (see above). The resulting PCR fragment was cut with XhoI and HindIII and subcloned in pGL3-basic opened with the same enzyme. **pRL-TK** (#402) encodes the *Renilla* luciferase.

### Cell Culture and Chemicals

The RasGAP^+/+^ mouse embryonic fibroblasts (MEFs; clone 12.78), the RasGAP^−/−^ MEFs (clone 12.64), and their derivatives stably expressing wild-type RasGAP or the caspase-resistant D455A mutant [11], [42] were maintained in DMEM (Invitrogen, catalogue n°61965) containing 10% newborn calf serum (Invitrogen, catalogue n° 26010-074) at 37°C and 5% CO_2_. BAD knock-out MEFs were cultured similarly. Mouse beta cells (MIN6) cells were maintained in modified DMEM complemented with 0.1 mM β-mercaptoethanol and 1 mM sodium pyruvate. HeLa cells were maintained in RPMI 1640 (Invitrogen catalogue n°61870) containing 10% newborn calf serum at 37°C and 5% CO_2_. Cisplatin (catalogue n°P4394) and rapamycin (catalogue n°R0395) were purchased from Sigma. Hexameric Fas ligand, resulting from the aggregation of 6 fusion proteins between Fas ligand and the Fc portion of IgG1 [43], was provided by Pascal Schneider (University of Lausanne). Staurosporine was purchased from Roche Diagnostics (Basel, Switzerland) (catalogue n°1055682).

### Antibodies

The anti-p70 S6 kinase antibody, the anti-raptor antibody, the anti-phospho Bad antibody sampler kit and the rabbit anti-survivin 71G4 monoclonal antibody were purchased from Cell Signaling Technology (catalogue n°9205, 2280, 9105 and 2808 respectively). The monoclonal anti-α tubulin antibody was purchased from Serotec, Oxford, UK (catalogue n°MCA77G). Secondary antibodies used for Western blotting were Alexa Fluor 680–conjugated anti-rabbit antibody (Molecular Probes, Eugene, OR; catalogue n°A21109) or IRDye 800–conjugated anti-mouse antibody (Rockland, Gilbertsville, PA; catalogue n°610-132-121). For immunohistochemistry the secondary antibody was Cy3-conjugated AffiniPure Fab fragment goat anti-rabbit IgG (catalogue n°711-165-152) and was purchased from Jackson ImmunoResearch Laboratories.

### Lentivirus

Recombinant lentiviruses were produced as described [44]. The minimal amounts of viruses inducing expression of the protein of interest in more than 95% of the cells (as assessed by immunofluorescence) were used.

### Transfection and Luciferase Assay

HeLa cells were transfected with Lipofectamine 2000 (Invitrogen) as described earlier [12]. MIN6 cells (3·10^5^) were co-transfected using Lipofectamine with 0.5 µg of mSurvivin promoter [−1297 to +13].gl3 or mSurvivin promoter [−209 to +13].gl3, 0.5 µg of a Renilla-encoding plasmid and 1 µg of either HA-hRasGAP[1–455](D157A).dn3 or mE2F1.sport6. The empty pcDNA3 or SPORT6.1 vectors were used as negative controls. Cells were harvested 36 hours after transfection and luciferase activity was measured using Dual-Luciferase® Reporter Assay (Promega). Signals were detected with GLOMAXTM 96 Microplate Luminoteter (Promega) and analyzed with Glomax version 1.7.0 program. Results were normalized with *Renilla* luciferase activity.

### RNAi

HeLa cells were plated in 6-well plates, with a starting density of 2·10^5^ cells per well. After an overnight incubation, the cells were transfected with 200 pmoles of siRNA for 7 hours using the calcium phosphate co-precipitation method [45]. siRNAs were synthesized by Microsynth (Balgach, Switzerland). The Raptor-directed siRNA sequence was 5′-GGACAACGGCCACAAGUA-3′ (nucleotides 2338–2355 found in the coding sequence of human Raptor transcript 1 mRNA; NCBI reference sequence: NM_020761.2). Cells were lysed 24, 48, and 72 hours after transfection for Western blotting analysis.

### RNA Extraction and Reverse Transcription

RNA was extracted by lysing cells with 500 µl TRI buffer (1.7 M guanidium thiocyanate, 0.1 M sodium citrate, 0.25% *N*-lauryl-sarcosyl sodium, 0.05 M β-mercaptoethanol, 0.1 M sodium acetate), followed by the addition of 200 µl of chloroform. The tubes were then vortexed, kept at room temperature for 5 minutes, and spun at maximal speed in an Eppendorf centrifuge for 15 minutes. After the transfer of the aqueous phase in a new tube, 500 µl of isopropanol was added and the solution was mixed by inversion approximately five times. The samples were then incubated o/n at −20°C. After spinning 20 minutes at maximum speed in an Eppendorf centrifuge, the upper phase (isopropanol) was aspirated; the pellet was washed twice with 800 µl 70% ethanol and dried 5–10 minutes at 50°C. The pellet was finally resuspended in 50 µl of water and RNA was quantitated at 260 nm. Half a microgram of RNA was mixed with 500 ng of random hexamers (Microsynth, Balgach, Switzerland), and water was added to reach a final volume of 11 µl. The samples were incubated 3 minutes at 70°C and then kept on ice. A mix (14 µl) containing 5 µl of 10 nM dinucleotide triphosphates (Promega catalogue n°U120D-123D), 0.5 µl of RNasin (Promega catalogue n°N211A), 5 µl of buffer 5×, 2 µl dithiothreitol, 0.5 µl Superscript reverse transcriptase, and 1 µl water (the 5× buffer, the dithiothreitol and the transcriptase are coming from the Superscript II reverse Transcriptase Kit; Invitrogen catalog n°18064-014) was added to the RNA-hexamer mix. After incubation at 39°C for 1 hour followed by 15 minutes at 70°C, the cDNA was diluted 1∶3 in water.

### Real Time PCR

Quantitative PCR assays were carried out on a real-time PCR detection system (iQ5; Bio-Rad) using iQ SYBR Green Supermix (Bio-Rad catalog n°170–8862) in which 500 nM primers and 1 µl of the cDNA template produced as described above were added (final volume of the reaction: 20 µl). The annealing temperature was 59°C. Melting curve analyses were performed on all PCR to rule out nonspecific amplification. Reactions were carried out in triplicate. The primers used were 5′-GCG GAG GCT GGC TTC A-3′ (forward primer) and 5′-AGA AAA AAC ACT GGG CCA AAT C-3′ (reverse primer).

### Propidium Iodide (PI) Staining and Flow Cytometer Analysis

HeLa (3·10^5^) cells were seeded in 3.5 cm dishes and infected with either an empty virus or a virus encoding an HA-tagged form of fragment N. Seventy-two hours after infection, cells were synchronized in G1-S phase with 400 µM of mimosine for 18 hours. Cells were released from mimosine blockage by changing the medium, and harvested at different time points. Cells were washed twice with 1× PBS and fixed with ethanol 100% for 15 minutes at −20°C. After fixation, cells were spun down at 2000 rpm at 4°C and washed once with 1× PBS. Cells were resuspended in 1 ml of PI staining buffer (100 mM Tris pH 7, 150 mM NaCl, 1 mM CaCl_2_, 0.5 mM MgCl_2_, 0.1% NP-40, 20 µg/ml RNAse A, 1 mg/ml propidium iodide diluted 1∶500) and put into sorter tubes. After 15 minutes incubation at room temperature in the dark, the cells were scanned using a Beckman Coulter FC500 flow cytometer with the following parameters: FSC 95 Volts-1 Gain, SSC 519 Volts-1 Gain, FL3 lin 313 Volts-1 Gain.

The population of cells to analyze was gated according to their size (FSC forward scatter) and granulosity (SCC side scatter) to exclude dead cells and clumps. Within this cell population cells were gated according to the peak of intensity versus the integrated signals to exclude debris and doublets. Ten thousand cells were analyzed per condition.

### Western Blot Analysis

Cells were lysed in monoQ-c buffer [12] and protein quantification was performed by the Bradford technique. Equal amounts of protein were migrated in a polyacrylamide gel and transferred onto a Trans-Blot nitrocellulose membrane (Bio-Rad catalogue n°10484060). Membranes were blocked with 5% non-fat milk and incubated over-night at 4°C with specific primary antibodies. Blots were washed with TBS/0.1% Tween 20, incubated with specific secondary antibodies and visualized with the Odyssey infrared imaging system (LICOR Biosciences, Bad Homburg, Germany). Quantification was performed using the Odyssey infrared imaging software.

### Apoptosis Measurements

Apoptosis in Hela cells was determined by scoring the number of transfected cells (i.e. cells expressing GFP) displaying pycnotic or fragmented nucleus [39]. Apoptosis in infected MEFs was similarly assessed but in all cells.

### Immunocytochemistry

HeLa cells were seeded in coverslips. Twenty-four hours later, coverslips were transferred to a clean dish and the immunocytochemistry was performed. Cells were fixed with 2% paraformaldehyde diluted in 1× PBS for 15 minutes at room temperature. After washing thrice with PBS, cells were permeabilized with 0.2% Triton X-100 diluted in 1× PBS for 10 minutes at room temperature. After washing with PBS, unspecific binding sites were blocked using DMEM culture medium complemented with 10% newborn calf serum. After 20 minutes at room temperature, the primary anti-HA antibody (1∶100 dilution) was added and incubated for 1 hour at room temperature in a dark and humid chamber. The secondary antibody was added after three washes in PBS for 1 hour at room temperature in a dark and humid chamber. After extensive washes in PBS, nuclei were stained with 10 µM Hoechst 33342 (Molecular Probes; catalogue n°H1399) for 10 minutes before mounting the slides in 0.1 g/ml Mowiol, 0.22% (v/v) glycerol, Tris 0.1 M pH 8.5, 0.1% diazobicyclo-octane. Mowiol was from Calbiochem (catalogue n°475904) and diazobicyclo-octane was from Fluka (catalogue n°33480).

### Mice and UV-B Irradiation

The RasGAP^D455A/D455A^ knock-in (KI) mice that cannot cleave RasGAP (C57BL/6N;129/SvEv-Rasa1^tm1Wid^) have been described earlier [10]. UV-B illumination was performed as described in reference [35].

### Immunofluorescence in Skin Sections

Paraffin sections were deparaffinized in two consecutive 5 minute long Xylene 100% baths and rehydrated by successive 2 minute long washes in ethanol 100%, 96%, 75% and 50%. Immunohistochemistry was performed as described [21]. Nuclei were stained with 10 µM Hoechst 33342 (Molecular Probes; catalogue n°H1399) for 10 minutes before mounting the slides in 0.1 g/ml Mowiol, 0.22% (v/v) glycerol, Tris 0.1 M pH 8.5, 0.1% diazobicyclo-octane. Mowiol was from Calbiochem (catalogue n°475904) and diazobicyclo-octane was from Fluka (catalogue n°33480). Quantitation of fluorescent positive cells was performed as previously described [46].

### Data Presentation and Statistics

Results are expressed as the mean ±95% confidence intervals (CI). The statistical tests used were one way ANOVAs unless otherwise stated. Normality of the data was verified with the Shapiro-Wilk test.

## Results and Discussion

To prevent cleavage of fragment N into the smaller N1 and N2 fragments, which can potentially generate confounding results, the experiments described below were performed with a form of fragment N that has its caspase-3 cleavage site destroyed [1], [12].

### mTORC1 and Fragment N-induced Protection

**Figure 1:**
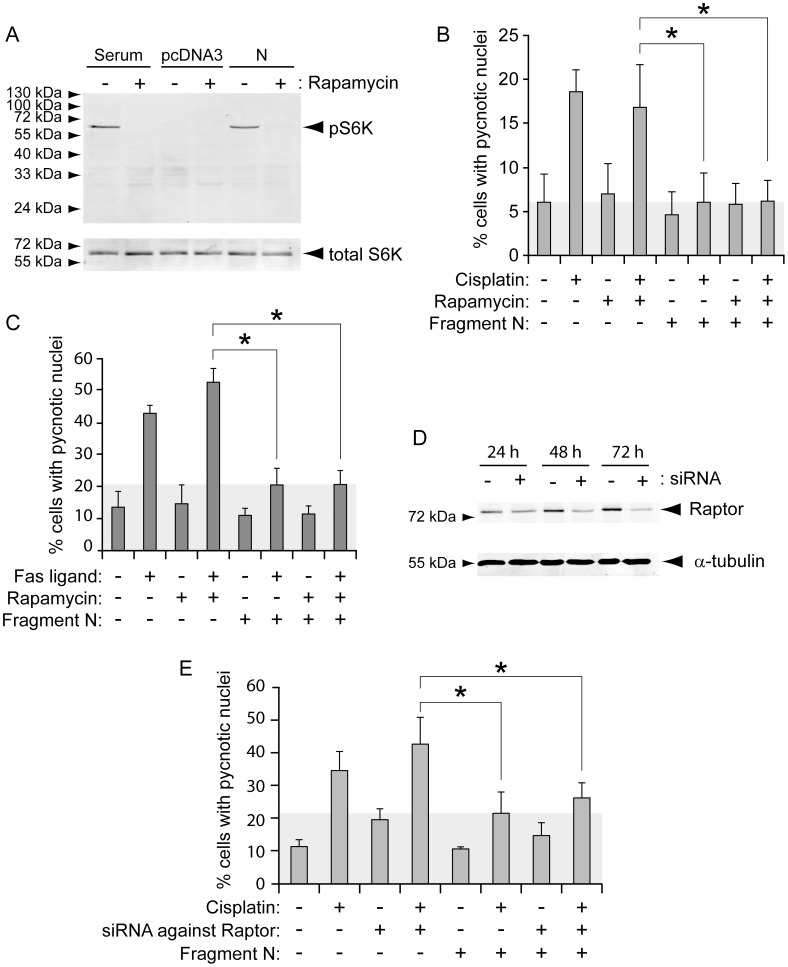
mTORC1 is not involved in fragment N-induced cell survival. HeLa cells were transfected with a GFP-expressing plasmid together with empty pcDNA3 or with pcDNA3 encoding the D157A caspase-resistant form of fragment N. Twenty-four hours later, the cells were incubated 90 minutes with 20 ng/ml rapamycin or with vehicle alone (DMSO). **A.** The cells were lysed after an additional 24-hour period and the extent of S6K phosphorylation was assessed by Western blot. **B-C**. Following the rapamycin pre-incubation period, the cells were treated or not with 10 µM cisplatin (panel B) or with 5 ng/ml Fas ligand (panel C) for 24 hours and apoptosis was scored (mean ±95% CI of 5 and 3 independent experiments, respectively, performed in monoplicate). **D**. HeLa cells were transfected or not with siRNAs directed at Raptor and one day later transfected with empty pcDNA3 or with pcDNA3 encoding fragment N. After an additional 24-, 48-, or 72-hour period, the cells were lysed to assess the levels of Raptor expression. **E**. Alternatively, one day following the pcDNA3 plasmid transfection, the cells were treated with cisplatin (30 µM) for 24 more hours and apoptosis was then scored (mean ±95% CI of 3 independent experiments performed in monoplicate). Asterisks denote statistically significant differences [one-way ANOVAs followed by pair-wise Dunn (Bonferroni) post hoc t tests].

Rapamycin, a macrolide antibiotic, has been widely used as a selective inhibitor of the mTORC1 complex and reported as an inducer of autophagy [47]. Depending on the cellular context and the concentrations of the drug used, rapamycin can either induce or inhibit apoptosis [48]. Rapamycin displays cytotoxic effects when used in micro-molar concentrations [49]–[51]. These studies highlight the impact of rapamycin dosage in cell survival outcome. In our experiments, we used 20 nM of rapamycin that did not induce, as expected, apoptosis (Figures 1B, 1C) but that was sufficient to fully block serum-induced phosphorylation of S6 kinase, an mTORC1 substrate (Figure 1A, first two lanes). This also indicates that mTORC1 is the sole kinase mediating S6K phosphorylation in serum-cultured HeLa cells. In starved conditions (Figure 1A, third lane), mTORC1 was no longer activated as indicated by the absence of S6K phosphorylation. As expected from its ability to stimulate Akt, fragment N activated mTORC1-dependent S6K phosphorylation (Figure 1A, last two lanes). However, the ability of fragment N to protect cells from cisplatin- or Fas ligand-induced apoptosis was unaffected by rapamycin (Figure 1B), suggesting that mTORC1 activation does not modulate fragment N-mediated protection. To further substantiate this point, we aimed to disrupt mTORC1 signaling by silencing Raptor, a protein of the mTORC1 complex [27]. Figure 1D shows that the strongest reduction in Raptor protein expression levels was achieved 72 hours after the siRNA transfection. We therefore assessed the ability of fragment N to protect cells against Fas ligand in which Raptor was silenced for 3 days. Figure 1E shows that Raptor silencing did not influence the ability of fragment N to inhibit Fas ligand-induced apoptosis. In conclusion, inhibition of the mTORC1 complex by rapamycin or via silencing of one of its components (Raptor) did not compromise the protective capacity of fragment N. Hence, mTORC1 does not appear to be an essential element targeted by fragment N to mediate its anti-apoptotic activity in cells.

### Phosphorylation of Bad does not Play a Major Role in Fragment N-mediated Protection

**Figure 2:**
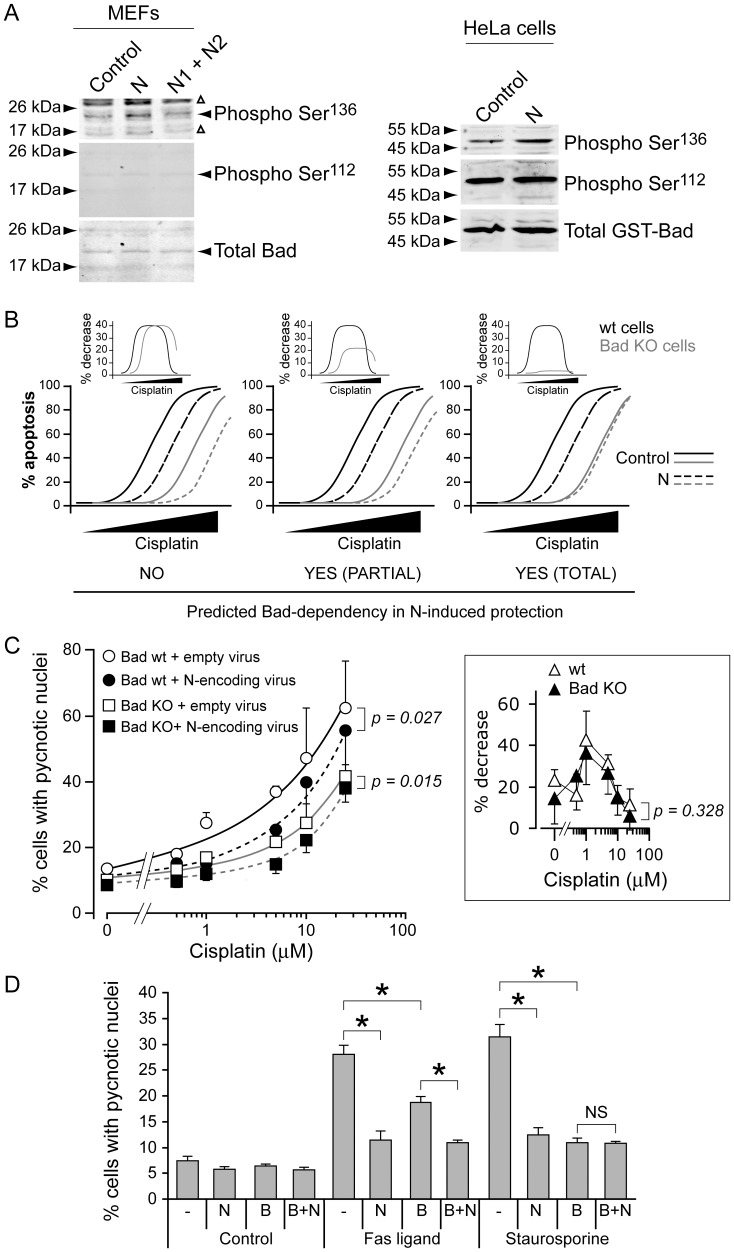
Bad modulation plays little role in fragment N-induced cell survival. **A.** MEFs were infected with an empty lentivirus or with lentiviruses encoding fragments N, N1, and N2, as indicated. Alternatively, HeLa cells were transfected with a plasmid encoding a GST-Bad fusion protein with either empty pcDNA3 or with pcDNA3 encoding fragment N. The cells were lysed 24 hours later. The extent of Bad phosphorylation was assessed by Western blot using antibodies specific for Bad phosphorylated on serine 112 or serine 136. **B.** Predicted apoptotic response in cells lacking or not Bad in the presence or in the absence of fragment N. See text for details. **C.** MEFs expressing or not Bad were infected with an empty lentivirus or with a fragment N-encoding lentivirus. Forty-eight hours later, the cells were incubated with the indicated concentrations of cisplatin. Apoptosis was assessed after an additional 24 hour-period. The inset depicts the reduction in the percentage of apoptosis induced by the expression of fragment N in wild-type or Bad KO cells at the indicated doses (using the values of the main figure). The results correspond to the mean ±95% CI of sextuplets derived from 4 independent experiments. Statistics were done by repeated measures ANOVA tests. **D.** MEFs were infected with an empty lentivirus (-), with a Bcl-2 (B)-encoding lentivirus, and with a fragment N (N)-encoding lentivirus, in the indicated combinations. Fourty-eight hours later, the cells were incubated with 30 ng/ml Fas ligand or 10 nM staurosporine. Apoptosis was assessed after an additional 24 hour-period. The results correspond to the mean ±95% CI of 4–8 independent experiments. Asterisks denote statistically significant differences [one-way ANOVAs followed by pair-wise Dunn (Bonferroni) post hoc t tests]; NS, not significant.

Bad is a pro-apoptotic protein that is blocked from triggering apoptosis by Akt-mediated phosphorylation on serine 136 [31]. Bad can be phosphorylated by other kinases, such as PKA, on serine 112 [52]. As expected from its ability to stimulate Akt, fragment N expressed in HeLa cells led to an increase in Bad phosphorylation on serine 136 but not on serine 112 (Figure 2A). Fragments N1 and N2, the fragment N cleavage products generated by high caspase-3 activity that are unable to stimulate Akt [11], did not induce Bad phosphorylation (Figure 2A).

The potential contribution of Bad in fragment N-mediated cell protection was assessed in MEFs derived from wild-type and Bad knock-out mice. Figure 2B displays the possible outcome of experiments using these cells expressing or not fragment N and stimulated with increasing concentrations of cisplatin. As Bad mediates part of the apoptotic response to cisplatin [53], reduced cisplatin-induced death is expected in Bad knock-out (KO) MEFs (orange lines). If Akt-induced Bad phosphorylation does not play any role in the protection mediated by fragment N, the latter should decrease the extent of apoptosis to similar extent in wild-type and Bad KO MEFs (Figure 2B, left graph). In other words, the maximal percent decrease in apoptosis induced by fragment N should be similar in both MEF types as shown in the inset above the left graph in Figure 2B. In contrast, if Bad phosphorylation is the sole mechanism by which fragment N protects cells, the sensitivity of Bad KO MEFs to cisplatin should be unaffected by the presence of fragment N (Figure 2B, right graph). In this case, for Bad KO cells, the difference between the apoptosis curves in the presence or in the absence of fragment N should be minimal (orange lines in the inset above the right graph in Figure 2B). The middle graphs in Figure 2B display a situation where Bad phosphorylation is partially contributing to fragment N-mediated cell protection. The actual experiment (Figure 2C) generated a pattern that corresponded to the scenario presented on the left of Figure 2B, suggesting that Bad plays no or only minimal role in fragment N-induced cell protection.

We reasoned that if Bad is not important for the capacity of fragment N to inhibit apoptosis, fragment N should be able to block cell death when the extrinsic pathway is activated, after Fas ligand stimulation for example, because this mode of death is not regulated by Bad. A potential caveat with this approach is that there is a possibility of a connection of Fas-induced death signaling to the intrinsic pathway via the cleavage of Bid [54]. This connection with the intrinsic pathway of apoptosis can however be blocked by over-expression of Bcl-2 [55]. Figure 2D shows that the stimulation of the mitochondrial intrinsic pathway activated by staurosporine in HeLa cells was fully prevented by Bcl-2 over-expression (compare the 9^th^ and 11^th^ bars). HeLa cells are type 2 cells as inhibition of the intrinsic pathway reduces apoptosis induced by Fas ligand (Figure 2D, compare the 5^th^ and 7^th^ bars); see also [56]. Importantly, the remaining apoptotic response can be fully blocked by fragment N (compare the 7^th^ and 8^th^ bars). This indicates therefore that fragment N can protect cells against a cell death stimulus in which pro-apoptotic Bcl-2 family members such as Bad play no role. Altogether, the experiments shown in Figure 2 support the notion that Bad is not a critical target of fragment N for its ability to protect cells.

### Fragment N does not Regulate Survivin

There is evidence that Akt can induce survivin expression [38], [57], [58]. As survivin may display anti-apoptotic activity in some conditions [36]–[38], [59], [60], it could be one of the main targets of fragment N that mediates its survival effects. We therefore assessed whether fragment N can regulate survivin by determining if it modulated its expression *in vitro* and *in vivo* and whether it affected survivin capacity to regulate cell division.

**Figure 3:**
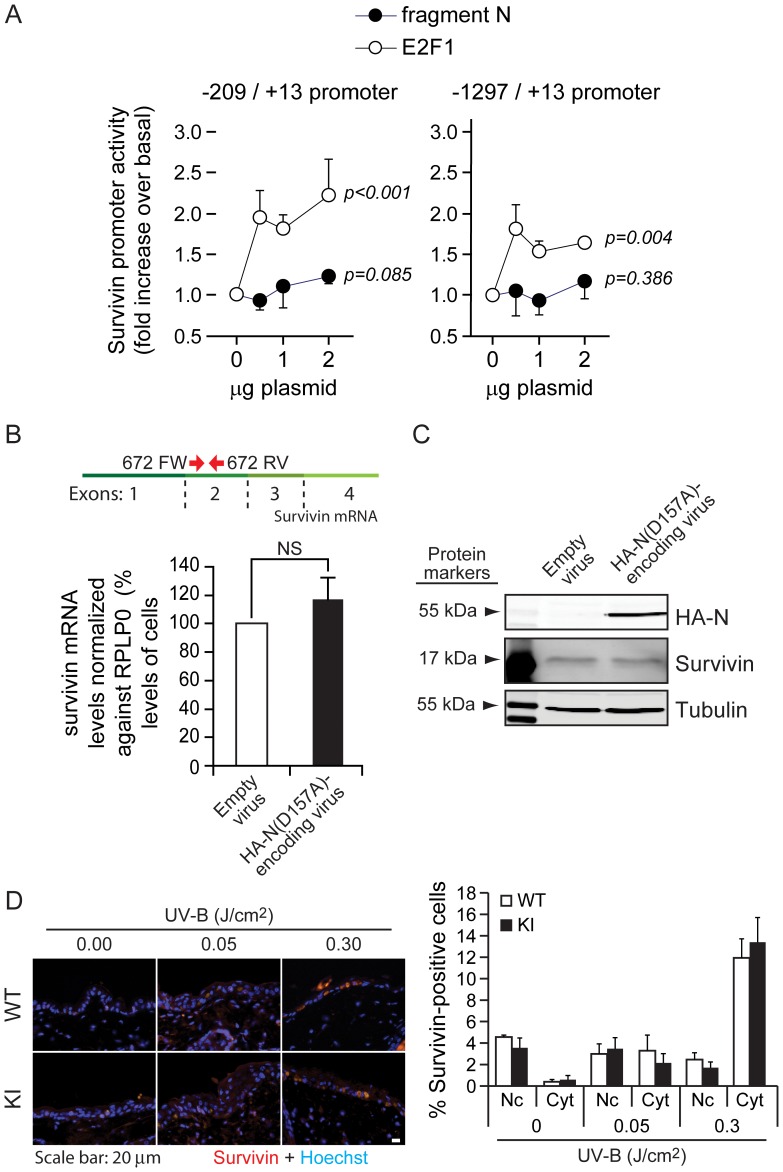
Fragment N does not regulate survivin expression. **A.** MIN6 cells were co-transfected with a luciferase expressing vector under the control of either a minimal promoter sequence allowing the transcription of survivin (left graph) or the entire survivin promoter sequence (right graph) with increasing amounts of fragment N- (closed circles) or E2F1- (open circles) encoding plasmids. The results correspond to the mean ±95% CI of 6 (left panel) and 3 (right panel) independent experiments performed in triplicate. Repeated measures ANOVA tests were performed to determine if there was a significant increase in luciferase activity induced by the E2F1- or fragment N-encoding plasmids (normality was verified with the Shapiro-Wilk test). **B–C**. MEFs were infected with an empty virus or with a lentivirus encoding the HA-tagged version of the D157A fragment N mutant. Survivin mRNA levels were analyzed 24 hours later by quantitative RT-PCR (panel B). The location of the 672FW and 672RV primers (red arrows), used for the amplification of the survivin mRNA, is depicted on top of the graph. Alternatively, cells were lysed and the protein expression of HA-fragment N and survivin was assessed by Western blotting (panel C). The results correspond to the mean ±95% CI of 3 independent experiments. **D.** Skins of mice were irradiated with low (0.05 J/cm^2^) and high (0.3 J/cm^2^) doses of UV-B light. Expression of nuclear and cytoplasmic survivin was assessed by immunofluorescence *in situ* (left panel). The quantitation shown on the right-hand side corresponds to percentages of keratinocytes displaying nuclear or cytoplasmic survivin (mean ±95% CI of 6 and 10 mice for the low and high UV-B dose exposure, respectively). No cells were found to display both cytoplasmic and nuclear survivin expression.

The effect of fragment N on survivin transcription was assessed by luciferase assay in which either the minimal or the entire sequence of the mouse survivin promoter was used. Fragment N expression in cells had no significant effect on either promoter activities (Figure 3A). As a positive control for this experiment, cells were transfected with an E2F1-expressing vector that is known to mediate survivin transcription [61] and this resulted, as expected, in an increase in survivin promoter activity (Figure 3A). To further confirm these results, real time PCR was performed to measure survivin mRNA in cells expressing or not fragment N. Figure 3B shows that fragment N did not induce an increase in the mRNA coding for survivin. Furthermore, survivin protein levels were not affected by fragment N (Figure 3C).

We have recently demonstrated that UV-B exposure of the epidermis leads to Akt phosphorylation in a caspase-3 and RasGAP cleavage-dependent manner [10]. Moreover, it has also been shown that survivin expression and relocalization to the cytoplasm, where it is supposed to induce its anti-apoptotic response [62], is induced in mouse skin in response to UV-B light [46]. Given that fragment N induces Akt in the epidermis of UV-B-irradiated mice [10] and that cytoplasmic survivin expression is augmented in this same tissue [46], we tested whether survivin expression in the skin is affected in knock-in (KI) mice that cannot generate fragment N because of a mutation in the first caspase-3 cleavage site of RasGAP. Control and KI mice were exposed to UV-B light 24 hours prior to biopsy and survivin levels were monitored in the skin by immunofluorescence. The percentage of keratinocytes expressing cytoplasmic survivin was increased by UV-B in a dose-dependent manner. This increase was similar in wild-type and KI mice (Figure 3D). This indicates that even though the epidermis of mice unable to generate fragment N is more sensitive to stress-induced apoptosis [10], it is still able to induce cytoplasmic survivin expression to levels that are observed in wild-type mice (Figure 3D). These results suggest that cytoplasmic survivin is not involved in fragment N-mediated protection, at least in mouse skin. Of note, the percentage of keratinocytes expressing nuclear survivin was not affected by UV-B light (Figure 3D).

**Figure 4:**
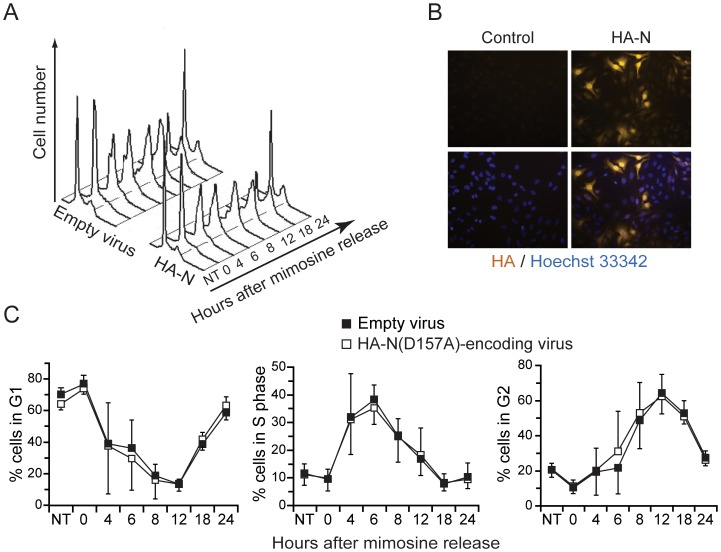
Fragment N does not affect cell cycling. HeLa cells, infected with an empty virus or with a lentivirus encoding the HA-tagged version of the D157A fragment N mutant, were synchronized at G1 by treatment with mimosine for 18 hours. The cells were then washed and cultured in fresh medium for the indicated periods of time. **A.** Representative histograms obtained at different time points after release from the mimosine block (NT, not synchronized cells). **B.** Immunocytochemistry-based detection (gold staining) of fragment N in the infected cells. The nuclei are colored in blue (Hoechst staining). **C.** Percentage of cells in each phase of the cell cycle as determined by PI staining (DNA content). Results represent the mean ±95% CI of 4 independent experiments.

Survivin is a protein with an important role in mitosis, with a peak of expression at G2/M phase and is therefore highly regulated in a cell cycle dependent manner [61], [63]. Even though fragment N does not appear to affect survivin levels, it may regulate its well-described function during mitosis. In order to study whether fragment N affects the cell cycle, cells expressing or not fragment N were synchronized in G1 using a mimosine block and then released from this block to resume cell cycling. Figure 4 shows that ectopic expression of fragment N did not alter the cell cycle of HeLa cells. There is thus no evidence that fragment N regulates survivin *in vitro* or *in vivo*.

In conclusion, the results presented in this work did not point to a crucial role of a given Akt effector in fragment N-mediated cell protection. Fragment N-mediated protection was not affected by mTOR inhibition or in BAD KO cells. Furthermore, survivin expression was neither modulated by fragment N nor was its function impaired in cells unable to generate fragment N upon stress. Possibly, fragment N relies on several Akt downstream targets to mount an efficient cell survival response. Alternatively, Akt effectors that we have not tested in the present work may fulfill most of the anti-apoptotic response induced by fragment N. Further studies need to be conducted to determine the exact contribution of Akt targets that mediate the capacity of fragment N to protect cells.
